# Molecular Diagnosis and Identification of Novel Pathogenic Variants in a Large Cohort of Italian Patients Affected by Polycystic Kidney Diseases

**DOI:** 10.3390/genes14061236

**Published:** 2023-06-08

**Authors:** Ersilia Nigro, Maria Amicone, Daniela D’Arco, Gina Sellitti, Oriana De Marco, Maria Guarino, Eleonora Riccio, Antonio Pisani, Aurora Daniele

**Affiliations:** 1 CEINGE—Biotecnologie Avanzate Scarl “Franco Salvatore”, Via G. Salvatore 486, 80145 Napoli, Italy; ersilia.nigro@unicampania.it (E.N.); darco@ceinge.unina.it (D.D.); 2 Dipartimento di Scienze e Tecnologie Ambientali, Biologiche, Farmaceutiche, Università della Campania “Luigi Vanvitelli”, Via Vivaldi 43, 81100 Caserta, Italy; 3 Unità di Nefrologia, Dipartimento di Sanità Pubblica, Università di Napoli “Federico II”, Via Pansini 5, 80131 Napoli, Italy; maria.amicone@unina.it (M.A.); oriana.demarco@unina.it (O.D.M.); eleonora.riccio83@gmail.com (E.R.); antonio.pisani@unina.it (A.P.); 4 Gastroenterology and Hepatology Unit, Department of Clinical Medicine and Surgery, University of Naples “Federico II”, Via Pansini 5, 80131 Naples, Italy; maria.guarino@unina.it; 5 Dipartimento di Medicina Molecolare e Biotecnologie Mediche, Università degli Studi “Federico II”, Via Pansini 5, 80131 Napoli, Italy

**Keywords:** polycystic kidney diseases, next-generation sequencing, ADPKD, ARPKD, genetic complexity

## Abstract

Polycystic Kidney Diseases (PKDs) consist of a genetically and phenotypically heterogeneous group of inherited disorders characterized by numerous renal cysts. PKDs include autosomal dominant ADPKD, autosomal recessive ARPKD and atypical forms. Here, we analyzed 255 Italian patients using an NGS panel of 63 genes, plus Sanger sequencing of exon 1 of the *PKD1* gene and MPLA (*PKD1, PKD2* and *PKHD1*) analysis. Overall, 167 patients bore pathogenic/likely pathogenic variants in dominant genes, and 5 patients in recessive genes. Four patients were carriers of one pathogenic/likely pathogenic recessive variant. A total of 24 patients had a VUS variant in dominant genes, 8 patients in recessive genes and 15 patients were carriers of one VUS variant in recessive genes. Finally, in 32 patients we could not reveal any variant. Regarding the global diagnostic status, 69% of total patients bore pathogenic/likely pathogenic variants, 18.4% VUS variants and in 12.6% of patients we could not find any. *PKD1* and *PKD2* resulted to be the most mutated genes; additional genes were *UMOD* and *GANAB*. Among recessive genes, *PKHD1* was the most mutated gene. An analysis of eGFR values showed that patients with truncating variants had a more severe phenotype. In conclusion, our study confirmed the high degree of genetic complexity at the basis of PKDs and highlighted the crucial role of molecular characterization in patients with suspicious clinical diagnosis. An accurate and early molecular diagnosis is essential to adopt the appropriate therapeutic protocol and represents a predictive factor for family members.

## 1. Introduction

Polycystic Kidney Diseases (PKDs) encompass a vast phenotypic spectrum of genetically and clinically heterogeneous disorders associated with different impacts on kidney function and with different treatment options [1,2]. 

Autosomal dominant polycystic kidney disease (ADPKD) is the most frequent among PKDs and the most common renal monogenic disease, affecting around 1 in 1000 individuals. ADPKD is a multisystemic disease with extrarenal manifestations, including polycystic liver disease (PLD), early onset hypertension, cerebral aneurysms and cardiovascular abnormalities and results the fourth cause of end-stage renal disease (ESRD) in adults [3,4,5]. Today, ADPKD is a condition treatable with tolvaptan, a selective vasopressin V2 receptor antagonist, and somatostatin analogues [6]. However, a differential diagnosis of ADPKD with other cystic renal diseases in certain cases remains crucial for early specific treatment.

Around 78% of ADPKD cases are linked to pathogenic variants in the *PKD1* gene and around 15% to pathogenic variants in the *PKD2* gene, while 7% of ADPKD cases remain genetically unresolved or are due to rare pathogenic variants in other genes [7,8]. Actually, about 2322 pathogenic variants in the *PKD1* gene and 278 in the PKD2 gene distributed without preferential high spots are listed in the ADPKD Mutation Database at Mayo Clinic (https://pkdb.mayo.edu/variants).

*PKD1* is a large complex human gene that spans 52 Kb on the 16p13.3 chromosome region and contains 46 exons; in addition, the 5′ to 33 exon region of *PKD1* is replicated in six pseudogenes that share 98% homology [9]. *PKD2*, located on the 4q21 chromosome region, is a gene constituted by 15 exons [10]. The *PKD1* and *PKD2* genes encode Polycystin 1 (PC1) and 2 (PC2), respectively, two membrane glycoproteins with the primary cilium as a likely functional site [11]. Another important genetic cause of renal-related morbidity is the autosomal recessive form of PKDs (ARPKD) caused mainly by mutations in the *PKHD1* gene, chromosome 6p12. *PKHD1* is among the largest human genes, with 86 exons assembled into a variety of alternatively spliced transcripts with the longest continuous open reading frame that encodes a multi-domain integral membrane protein (fibrocystin/polyductin) of unknown function [12]. The *PKHD1* mutations are also scattered throughout the gene and most of them are unique to single families [13].

The genetic heterogeneity of PKDs complicates the pathophysiology and the possibility of performing a precise and early diagnosis [14,15]. To further hamper the genetic diagnosis, it should be considered that (a) several other genetically inherited conditions have been associated with the formation of cysts in the kidney, and (b) there is a significant phenotypic and genotypic overlap between PKDs and polycystic liver diseases (PLD).

Genetic testing for PKD is a rapidly increasing area: applying PKD molecular testing in the clinical context could allow for diagnosis in suspected cases with no apparent family history or with equivocal imaging findings, improving patient care through disease prognostication, guide treatment, clinical trials and genetic counseling.

In this context, Next-Generation Sequencing (NGS) technology has upgraded the possibility to perform molecular diagnosis as well as to understand the genetic mechanisms linked to PKD conditions [16,17,18,19,20]. In this scenario, and based on literature data, we developed an NGS panel that includes 63 genes linked to PKDs to explore the genetic basis of the heterogeneous spectrum of PKD-like phenotypes in a large group of 255 Italian patients with suspicion or clinical diagnosis of PKD. We also performed Sanger sequencing of exon 1 of the *PKD1* gene and MPLA analysis on patients whose pathogenic variants were not identified.

## 2. Materials and Methods

### 2.1. Patient Recruitment, Clinic and Biochemical Evaluation

In total, 255 consecutive patients were recruited from Nephrology Unit, Department of Public Health, “Federico II” University, Naples, and Hospital outpatient clinic, “Federico II”, Naples. All patients were 18 years of age or above, able to provide informed consent and without previous genetic results related to PKD. Patients included were either with a positive family history of PKD and met the unified criteria for ultrasonographic diagnosis of PKD [5] or were without PKD family history but had confirmed renal cysts through imaging as per the unified criteria for ultrasonographic diagnosis of PKD. A 10 mL blood sample was collected in EDTA tubes from each enrolled patient. Genomic DNA was isolated from each blood sample using the MagPurix Blood DNA Extraction kit (Zinexts Life Science Corp., New Taipei City, Taiwan). The study was approved by the local ethics committee and complied with the guidelines of the Declaration of Helsinki. Informed written consent was obtained from each patient.

### 2.2. NGS Custom Panel Design and Panel Content

Molecular testing was carried out by analyzing a panel of target genes through an NGS-based procedure. For the selection of genes (*n* = 63) included in the panel, we relied on updated data from the literature together with the advice received from nephrologists [16,17]. The complete design of the PKDs panel is available in Supplementary Table S1. This panel includes several classes of genes involved in (i) folding, trafficking or degradation of kidney proteins; (ii) the protection of kidney epithelial cells; (iii) renal tubulogenesis; (iv) primary cilia formation and maturation; and (v) related syndromes. For each gene, we analyzed the coding regions, 25 bp in each of the intronic boundaries. 

### 2.3. DNA Isolation and NGS Library Preparation and Sequencing

DNA reference samples (*n* = 26) were obtained from the hospital “Casa Sollievo della Sofferenza, Istituto di Ricovero e Cura a Carattere Scientifico”, San Giovanni Rotondo, Foggia, Italy, and from CEINGE-Biotecnologie Avanzate “Franco Salvatore”, Napoli, Italy. Genomic DNA (gDNA) was isolated using the Nucleon BACC3 Genomic DNA Extraction Kit (GE Healthcare, Life Sciences, Chicago, IL, USA) according to the manufacturer’s instructions. The quality of DNA samples was assessed by the TapeStation system (Agilent Technologies, Santa Clara, CA, USA); a DNA integrity number (DIN) >6 was considered suitable for NGS analysis. DNA quantity was evaluated through the NanoDrop 2000c spectrophotometer (Thermo Fisher Scientific, Waltham, MA, USA) and by using Qubit dsDNA BR and HS assays kits (Life Technologies, Carlsbad, CA, USA).

DNA molecular analysis was performed using the above-mentioned NGS panel. HaloPlex technology (Agilent, Santa Clara, CA, USA) was used for library preparation. In detail, each genomic DNA sample was fragmented using a pool of restriction enzymes. The obtained fragments were enriched with hybridization with the custom capture probes, and then purified and PCR-amplified to obtain a DNA library or sample (total probes: 42,817; total probes size: 415.055 kbp; each probe was 120 mer). During this procedure, each genomic DNA sample was univocally tagged with a barcode sequence to allow for sample multiplexing during the subsequent sequencing step. The custom design of our probes was realized using the web-based SureDesign application. A total of 50 ng of gDNA was processed through the SureSelectQXT Target Enrichment system (Agilent Technologies, Santa Clara, CA, USA) for Illumina multiplexed sequencing. Briefly, gDNA was enzymatically fragmented and adaptor-tagged to obtain a pool of fragments that were amplified by PCR reaction. Then, the prepared DNA library amplicons were hybridized to the capture custom library, made up of 63 selected genes, and purified by streptavidin-coated magnetic beads. The captured, target-enriched DNA library was amplified by PCR reaction by using dual index primers, which allowed us to univocally barcode each sample. Finally, SureSelect-enriched dual-indexed NGS samples were pooled together for multiplexed sequencing. The sequencing reactions were carried out on the MiSeq instrument (Illumina, San Diego, CA, USA) using a flow cell micro, running 6/8 samples for each sequencing run to obtain an average coverage of about 100× (>90% of the analyzable target regions were covered by at least 50×).

### 2.4. NGS Data Analysis

The Alissa Align & Call v1.0.2.10 tool (Agilent Technologies, Santa Clara, CA, USA), using the genome build hg38 as a reference, was used to perform alignments, variant calling and quality filtering. The median QV bases used in variant calling was 39, with an average read length of 141 bp. Variant filtering and interpretation were performed using Alissa Interpret v5.2.6 CE IVD software (Agilent Technologies, Santa Clara, CA, USA), using GRCh38.p2 and annotation sources such as 1000 Genomes (Phase 3 release v5, 10 September 2014, including GRCh38 data), ClinVar (NCBI ClinVar October 2019), DGV (Database of Genomic Variants, version 15 May 2016), ESP6500 (variants in the ESP6500SI-V2 dataset of the exome sequencing project, annotated with SeattleSeqAnnotation137), ExAC (ExAC release 1.0—including GRCh38 from lift over data) and OMIM (OMIM, version 25 October 2019). To define mutations as novel, we also checked for their presence in two large public sequence databases: gnomAD (release 2.0.2, available at gnomad.broadinstitute.org; *n* = 15.496 diploid genome sequences and *n* = 123.136 diploid exome sequences) and BRAVO (powered by TOPMed Freeze5 on genome reference consortium human build 38 patch release 12, available at https://bravo.sph.umich.edu/freeze5/hg38/; *n* = 62.784 diploid whole-genome sequences).

### 2.5. Sanger Sequencing

All the variants considered, such as pathogenic, likely pathogenic and VUS, were confirmed by Sanger sequencing from genomic DNA extracted from the second patient’s blood sample. PCR amplification was carried out on a 2720 Thermal Cycler (Applied Biosystems Inc., Foster City, CA, USA) or VeritiPro Thermal Cycler (Applied Biosystems Inc., Foster City, CA, USA). Next, direct sequencing was performed using a 3730 DNA Analyzer (Applied Biosystem, Foster City, CA, USA). In particular, for the exons 1–46 of *PKD1*, we performed a nested PCR of the region of interest from LR amplicons before proceeding to Sanger sequencing. For the VUS and/or LP variants in *PKD1* gene with a low read depth (<20%), we performed targeted genotyping using specific primers (designed on the canonic gene or on the pseudogenes) to confirm that the functional *PKD1* gene was amplified and sequenced rather than the pseudogene. Sanger sequencing of exon 1 of the PKD1 gene was performed as previously described only on those patients negative for pathogenic variants (primers are available on request) [18].

### 2.6. Multiplex Ligation-Dependent Probe Amplification (MLPA)

After NGS procedure, genomic DNA from patient samples without variants classified as pathogenic, likely pathogenic or VUS was analyzed by MLPA for the detection of large gene rearrangements using the commercial kits SALSA MLPA P351 *PKD1* and SALSA MLPA P352 *PKD1*-*PKD2* probemix (MRC-Holland, Amsterdam, The Netherlands). Successively, for those patients without positive results in *PKD1*-*PKD2* SALSA MLPA, we proceeded to analyzing the *PKHD1* gene for the detection of large gene rearrangements using the commercial SALSA MLPA P341-B4 *PKHD1* mix 1 and P342-C1 kits *PKHD1* mix 2 probemix kits (MRC-Holland, Amsterdam, NL).

PCR product analysis was carried out on a 3730 DNA Analyzer and the electropherograms were visualized by Coffalyzer software version 130202.2357 (MRC Holland, Amsterdam, The Netherlands). The assays were performed according to the manufacturer’s instructions.

### 2.7. Variant’s Pathogenicity Predictions

Bioinformatics predictions of the variant’s effects were performed using the PolyPhen-2 (http://genetics.bwh.harvard.edu/pph2/) and Clinvar tools (https://www.ncbi.nlm.nih.gov/clinvar/). Further predictions were assessed with the Mutation Taster tool (http://www.mutationtaster.org) and VarSome website (https://varsome.com/variant/hg38). All software were used with their default parameters. Variant classification was performed according to American College of Medical Genetics and Genomics (ACMG) guidelines [21]. Variants were classified into five-tier categories: pathogenic, likely pathogenic, variants of uncertain significance (VUS), likely benign and benign.

The standard gene variant nomenclature, according to Human Genome Variation Society (http://www.hgvs.org/mutnomen) and corrected by https://mutalyzer.nl, was used. Information on protein structure and domains was obtained by consulting Uniprot (http://www.uniprot.org) and the PFam web resources (http://pfam.xfam.org/).

## 3. Results

In this study, we analyzed a large cohort of 249 Caucasian and 6 non-Caucasian patients affected by PKDs through a targeted NGS gene panel consisting of 63 genes related to PKD genetic diseases; 225 patients were unrelated and 30 were related. We found 167 patients bearing pathogenic/likely pathogenic variants in dominant genes and 5 patients with recessive genes of heterozygous or homozygous status; in 4 patients, we found only one pathogenic/likely pathogenic recessive variant. A total of 24 patients had a VUS variant in dominant genes while 8 patients had a variant in recessive genes; in 15 patients we found only one VUS variant in recessive genes. Finally, in 32 patients, both NGS and MLPA did not reveal any pathogenic mutation. The complete list of patients and genes involved is reported in Table 1.

**Figure 1:**
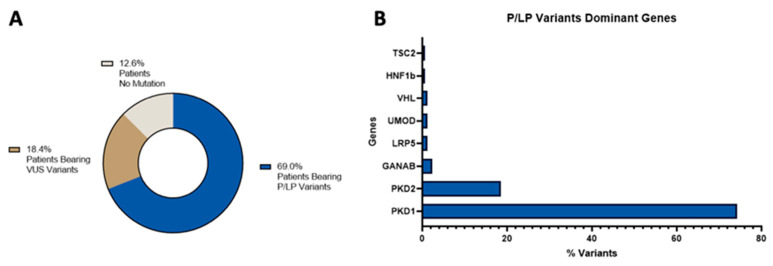
Distribution of the molecular diagnostic status (panel **A**) and of the pathogenic/likely pathogenic variants in dominant genes (panel **B**).

Regarding the global diagnostic status distribution, 69% of the total identified variants in our cohort of patients were pathogenic/likely pathogenic variants (Supplementary Tables S2 and S4), 18.4% were VUS variants (Supplementary Tables S3 and S5), and in 12.6% of patients we could not find any variants, see Figure 1, panel A. The majority of those pathogenic/likely pathogenic variants were found in dominant genes and, when we analyzed the involved genes, we found that, as expected, PKD1 and PKD2 were the most mutated ones, see Figure 1, panel B.

Nonsense and missense mutations were the most frequent pathogenic/likely pathogenic variants type within *PKD1*, accounting for 37.19% and 20.66%, respectively, of total *PKD1*-mutated patients; interestingly, among *PKD2*-mutated patients, the nonsense mutations represented the most frequent mutations (60% of total pathogenic/likely pathogenic *PKD2* variants). Phenotypically, we observed that *PKD2*-mutated patients showed an eGFR of 56.75 vs. 73.76 *PKD1* patients (*p* = 0.028). However, the age at diagnosis of *PKD1* patients (birth–64 years old, median 25) was lower than *PKD2* (20–60 years old, median 42) suggesting that *PKD2* patients are diagnosed at an older age compared to *PKD1*.

**Figure 2:**
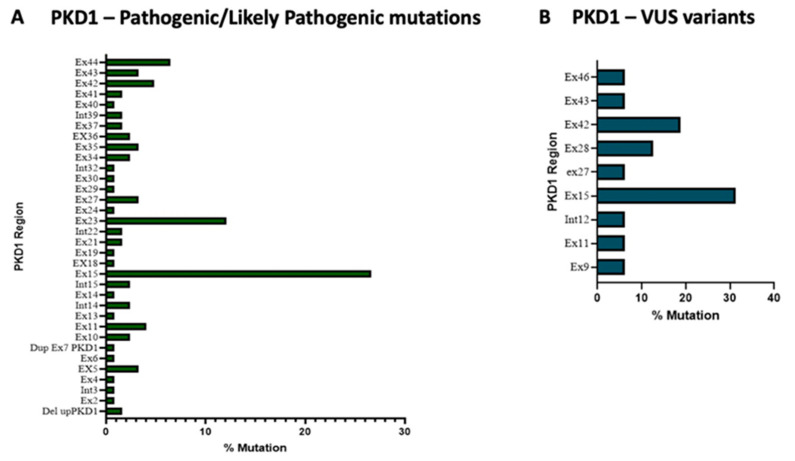
Exon’s pathogenic/likely pathogenic variants (panel **A**) and VUS variants frequency in *PKD1* gene (panel **B**).

We identified, in the *PKD1* gene, 83 different pathogenic/likely pathogenic variants and 16 VUS variants were found (see Supplementary Tables S2 and S4), 56% of the former were located in the exons 15, 23 and 45 (Figure 2, panel A). It can be noticed that exon 15, together with 5, 13 and 14, within *PKD1* encodes for the PKD globular domain of the Polycystin-1 (PC1) protein that represents a crucial structural and functional element of PC1 being involved in interactions with other proteins (cell–cell or cell–matrix interactions). On the other hand, the exons 23 and 44 were also frequently found to be mutated. These two latter exons are involved in the production of the PC1 C-terminal cytoplasmic tail, a fundamental domain for the interaction between PC1 and PC2 proteins, both crucial in renal disease progression.

Regarding the VUS variants within *PKD1*, exons 15 and 42 are the most frequently mutated, accounting for 44.3% of total variants (Figure 2, panel B).

**Figure 3:**
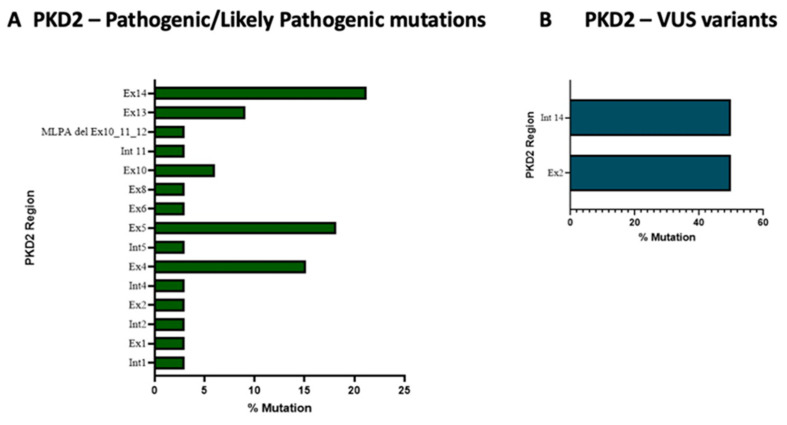
Exons’ pathogenic/likely pathogenic variants (panel **A**) and VUS variants’ frequency in *PKD2* gene (panel **B**).

Nineteen different pathogenic/likely variants and two VUS variants were found within the *PKD2* gene (Supplementary Tables S2 and S4). Exons 5 and 14 of *PKD2* accounted for the majority of pathogenic/likely pathogenic variants (41.93%) (see Figure 3, panel A); interestingly, exon 14 encodes the COOH-terminal region through which the transmembrane glycoprotein polycystin-2 (PC2) interacts with PC-1. Regarding the VUS variants within *PKD2,* we found only two variants in exon 2 and intron 14 (see Figure 3, panel B).

Among *PKD1*-positive patients, we found a family bearing a likely pathogenic variant in *PKD1* (c.10549G>T) together with a concomitant variant in the α-galactosidase A gene (*GLA*) (c.868A>C). Deficiency of the lysosomal GLA enzyme causes Fabry disease (OMIM: 301500), a rare X-linked hereditary disease (incidence 1:117.000 live births). The first patient to come to our attention was a 31-year old female patient with renal cysts and an eGFR of 92: NGS analysis revealed the presence of both the above-mentioned mutations. Successively, two relatives (one brother and a sister) were analyzed: the brother had both variants in *GLA* and *PKD1* with a moderate renal impairment plus a cardiac and skin impairment from Fabry disease, while the sister had the *PKD1* variant only. NGS analysis of the mother (with normal renal function) revealed that she is carrier of the GLA mutation. The father is *PKD1*-mutated with a severe PKD clinical phenotype. 

**Figure 4:**
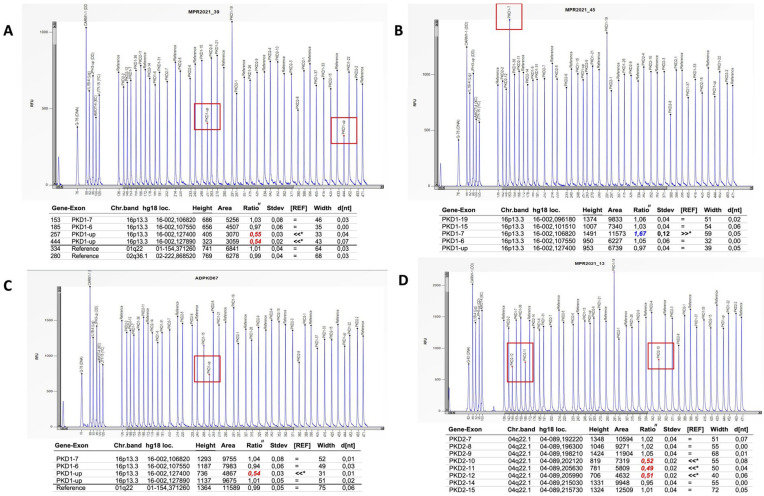
MPLA analysis has revealed two *PKD1* deletions (panels **A**,**C**), one *PKD1* duplication (panel **B**) and one deletion in *PKD2* gene (panel **D**).

For those patients without variants classified as pathogenic, likely pathogenic or VUS, we proceeded by analyzing exon 1 by Sanger sequencing and successively analyzing large gene rearrangements by MLPA (Figure 4). No mutations were found in exon 1. MPLA analysis identified three patients with large rearrangements in *PKD1*: two with large deletions (Figure 4, panels A,C) and one with duplication (Figure 4, panel B). In addition, one patient had a large deletion in *PKD2* (Figure 4, panel D).

The genotype description of the five recessive patients is shown in Table 2. Four of them reported two variants in *PKHD1* while one patient was homozygous for *MAPKBP1*. Two *PKHD1* patients and the *MAPKBP1*-positive patient bore pathogenic/likely pathogenic variants, while the other *PKHD1* patients carried VUS variants. It can be noticed that, within the entire cohort of patients, the *MAPKBP1*-positive patient was the only homozygous genotype we found. The mean eGFR of ARPKD patients was 62 vs. 72.3 for ADPKD ones.

Trans-heterozygous patients with two variants in different recessive genes are reported in Table 3. Only two patients carried pathogenic mutations, while in the other patients we could only identify VUS variants.

NGS analysis revealed that 101 patients had truncating mutations in the *PKD1, PKD2, PKHD1* and MA*PKBP1* genes. The mean e-GFR of patients bearing truncating mutations was 62.97 (73.58 for *PKD1* patients, 52.35 for *PKD2* patients, 67 for *PKDH1* patients and 9 for *MAPKBP1* patients, respectively) vs. 70.42 for all remaining patients (Table 4). Of note, all truncating mutations are predicted to be pathogenic or likely pathogenic, confirming that these mutations strongly affect protein structure and function.

We found 37 not previously reported pathogenic/likely pathogenic alterations: 31 in *PKD1*; 4 in *PKD2*; 1 in *UMOD*; and 1 in *MAPKB1* genes (see Table 5). Of them, 16.2% were missense mutations, 45.9% were frameshift and 10.8% were splicing. In addition to those variants, we also found 15 VUS novel variants whose clinical meaning needs to be confirmed by further analyses. 

## 4. Discussion

In this study, we analyzed a large cohort of 255 Italian PKD patients through a targeted NGS gene panel composed of 63 genes related to PKD genetic diseases. We found 167 patients carrying pathogenic/likely pathogenic variants in the dominant genes. Five patients had pathogenic/probably pathogenic variants in the recessive genes: four in the heterozygous state and one was homozygous in the *MAPKBP1* gene. Finally, in four patients, we found only one pathogenic/likely pathogenic variant. As regards the VUS variants, 24 patients presented a VUS variant in the dominant genes while 8 patients in the recessive genes; in 15 patients we found only one VUS variant in the recessive genes. Finally, in 32 patients, both NGS and MLPA analyses did not reveal any variants.

Genetics is presently rarely used for routine diagnostics in PKDs because there are many issues hampering the genetic diagnosis of these diseases: the large size, the high genetic complexity and the lack of mutational hotspot in the causative genes. However, in recent years, NGS technology has proven to be an effective alternative to conventional and unconventional techniques in PKDs [36,37,38]. Here, we described the design and development of an NGS panel (PKDs panel) to simultaneously screen the coding regions (CDS) of the genes together with 25 bp of regions adjacent to both the 5′ and 3′ of the 63 genes. With the NGS technique plus Sanger sequencing and MLPA, we describe here a methodological approach able to perform the molecular diagnosis of about 70% of patients with clinical evidence of PKD.

Considering the Italian population, previous studies analyzed ADPKD patients, reaching an overall detection rate of maximum 80% [7,16,39,40]. Another Italian group considered 119 individuals with inherited kidney diseases (polycystic and non-polycystic), testing 115 genes causing renal diseases and identifying the disease-causing variants in 51.5% and 40% of polycystic and non-polycystic individuals, respectively [40]. All of these data suggest that early clinical differentiation into cystic and non-cystic kidney disease is essential for best outcomes [41].

However, in cases of unclear or negative family anamnesis, on the other hand, it may be necessary to modify the clinical diagnosis, essentially based on imaging, following the genetic diagnosis; in fact, the same clinical phenotype may be due to mutations present in other PKD-associated genes. Therefore, knowing the genotype becomes very important in these cases to make the right therapeutic decisions.

In fact, for several patients (both affected by recessive forms as well as affected by dominant forms, but carriers of mutations in genes other than *PKD1*–*2*), conservative or target therapy (tolvaptan/octreotide) is not appropriate and/or even ineffective. Therefore, a wrong clinical diagnosis could potentially create a serious delay in the correct therapeutic approach, preventing personalized patient management.

Here, we identified in the *PKD1* gene 83 different pathogenic/likely pathogenic variants and 16 VUS variants, 56% of which were located in exons 15, 23 and 45. It can be seen that exon 15, together with 5, 13 and 14, within *PKD1* encodes for the PKD globular domain of the Polycystin-1 (PC1) protein that represents a crucial structural and functional element of PC1 being involved in interactions with other proteins (cell–cell or cell–matrix interactions). On the other hand, exons 23 and 42 were also frequently mutated in our patient population. These two latter exons are involved in the production of the PC1 C-terminal cytoplasmic tail, a fundamental domain for the interaction between PC1 and PC2 proteins, both crucial in renal disease progression [42]. 

In all patients found to be mutation-negative for the genes included in the NGS panel, we performed Sanger sequencing of PKD1 exon 1 (to obtain better and more complete sequencing) as well as MLPA analysis. This latter evaluation led to the identification of one large duplication and two large deletions in three patients with no mutations identified at the NGS procedure. Such data are in accordance with the frequency of large rearrangements reported by Carrera et al. [39]. From our analysis, it is interesting to outline that we found two patients—belonging to the same family—bearing a likely pathogenic mutation in *PKD1* and a concomitant likely pathogenic mutation in the *GLA* gene. In detail, the family consists of the mother, asymptomatic with normal renal function, carrying the mutation in the GLA gene; the father, characterized by a severe renal phenotype, carrying the PKD1 mutation; and the three children—one daughter, with a normal kidney function, carrying the PKD1 mutation, a second daughter carrying both PKD1 and GLA mutations with normal renal function, mild proteinuria and absence of symptoms of Fabry disease, and a third brother carrying both PKD1 and GLA mutations, with moderate renal insufficiency along with cardiac and skin impairment from Fabry disease. 

Based on the current clinical features, it is not possible to define whether there is a worsening of renal function in the male patient carrying both mutations, since they are not comparable to those of females. To our knowledge, only one published paper reported a similar genetic status [43]. The authors described a 60-year-old male patient who was diagnosed with Fabry disease at 34 years of age and a VUS variant in *PKD1*. In accordance with our observation, the patient fulfilled the clinical criteria for PKD. This observation further supports the importance of the genetic analysis of renal cystic patients through the examination of a large panel of PKD-associated genes. The presence of this patient in the PKD population confirms a precise indication of the importance of including the *GLA* gene in an NGS panel for the genetic diagnosis of PKD. Indeed, the formation of renal cysts in Fabry disease patients may represent an undiagnosed case of PKD [44]. 

To date, 278 mutations have been found in *PKD2* (https://pkdb.mayo.edu/variants) and here we observed 19 different pathogenic/likely variants and 2 VUS variants were found within the PKD2 gene (Supplementary Tables S2 and S4). The exons 5 and 14 of PKD2 accounted for the majority of pathogenic/likely pathogenic variants (41.93%). Interestingly, the exon 14 encodes the COOH-terminal region through which the transmembrane glycoprotein polycystin-2 (PC2) interacts with PC-1. In particular, PC-2 forms homotetrameric transient receptor potential channels that regulate the intracellular calcium concentration from the endoplasmic reticulum membrane and the primary cilium membrane [45]. The molecular changes in the interaction between *PKD1* and *PKD2* genes increase cell proliferation and fluid secretion, mechanisms at the basis of cyst formation [45]. It is therefore quite relevant that exon 14 is the main site of the causative mutations of *PKD2*. The MLPA analysis of PKD2 led to the identification of one large deletion. 

Interestingly, we found two unrelated ADPKD patients with pathogenic/likely pathogenic variants in *LRP5* and two patients with *LRP5* VUS variants. *LRP5* is a co-receptor strongly expressed in both the liver and kidneys and involved in the Wnt signaling pathway, whose disruption may predominantly lead to polycystic liver disease [46,47,48]. *LRP5* was first reported in relation to autosomal dominant polycystic liver disease with or without kidney cysts in 2014 [46]. Some expression studies have demonstrated *LRP5* expression in liver tissue [46,47,48]. In addition, some studies in the literature have reported that, in up to 94% of patients, *LRP5* variants may render ADPKD patients more susceptible to the development of polycystic liver disease [46,47,48]. Furthermore, there are functional and animal studies that support that *LRP5* variants may contribute to hepatic and renal cystogenesis.

However, overall, there is limited and unconvincing evidence to support a gene–disease relationship between *LRP5* and autosomal dominant polycystic liver disease with or without kidney cysts. Although more evidence is needed to support a causal role for polycystic disease, evidence has emerged that contradicts the gene–disease relationship. 

In this scenario, our data are quite interesting considering that the clinical picture of our patients correlates with cystic kidney disease, suggesting that the *LRP5* gene, in addition to polycystic liver disease, could potentially play a role in PKD predisposition.

Another gene causative of ADPKD is *GANAB* [49]. In this study, we identified the missense c.2087 G>A mutation (p.Arg696Gln) previously described (https://varsome.com/variant/hg38/ganab%20c.2087G%3EA?annotation-mode=germline) that co-segregates with the disease in four affected family members. The patients in this family suffered from both polycystic kidney as well as liver disease. In our cohort, 101 patients out of 257 had truncating mutations in *PKD1, PKD2, PKHD1* and *MAPKBP1*. The mean e-GFR of those patients was 62.97 vs. 70.42 for all other patients with other types of mutation, confirming that truncating mutations are associated with a more severe phenotype. In accordance with our data, both Heyer et al., and Hwang et al., reported that truncating *PKD1* mutations (frameshift, splicing and nonsense) have a more severe disease prognosis with lower eGFR [50,51,52,53].

Regarding the recessive form of the disease, *PKHD1* resulted to be the most mutated gene, representing 34.61% of total alleles: four patients bore the canonical heterozygous genotype, while in four patients we were able to identify a single mutation in heterozygosity, suggesting that some variants in *PKHD1* might cause ADPKD as well as ADPLD, as suggested by literature data [54,55]. However, the identification of a single mutation in *PKHD1* might also be due to the lack of identification of the second causative variant. In addition, family members should be recruited to deepen understanding of the pathological role of such mutations.

Thirty-seven not previously reported pathogenic or likely pathogenic variants in four different genes were identified. Moreover, the elevated number of not previously reported variants confirms the elevated molecular heterogeneity of the disease within our Italian PKD population, whereas previous reports described novel mutations only in a few genes [16]. Finally, we identified only one VUS variant in heterozygosity in 15 patients. It can be noted that the pathogenicity of VUS variants, as well as the dominant or recessive transmission of some genes and genetic variants, rapidly evolve through additional studies; therefore, some VUS will likely be reclassified as disease-causing and more monogenic causes of kidney disease will be identified. In light of this possibility, the rate of positive findings of the study may likely be an underestimate. The missing mutations in heterozygous subjects and in patients without identified mutations could be located in deep intronic or other regulatory regions distant from the splice donor and acceptor sites that have not been screened so far; alternatively, causative mutations could reside in other genes not analyzed in the present study and, more importantly, still not identified as genes involved in cystic renal disease. It should be considered, indeed, that the molecular pathways underlying cystic formation in the kidneys are quite complex and involve hundreds of proteins. In addition, patients without evidenced mutations should be considered not only as molecular undiagnosed patients but also as patients that, although phenotypically adherent to the PKD picture, need to be considered as different subjects requiring a different clinical management [38,56,57]. For those patients, further studies are required to deepen the germline and somatic genetic background—also possibly including additional genes in the analysis [58].

The limitations of this study include a lack of some clinical information and a clinical follow-up, mainly due the non-compliance of most families. Another limitation of the study may be the design of the gene panel that, although inclusive of numerous genes related to polycystic disease, may not contain additional genes potentially responsible for PKD. Despite these limitations, our results confirm that the NGS-based panel approach is a useful first-line tool for PKDs diagnosis. In the near future, as more patients are investigated, the information provided by such panels will continue to grow and improve understanding of these complex pathologies and hopefully improve the efficiency of such investigations.

Furthermore, thanks to genetic analysis, it has been possible to carry out diagnoses in complex families (i.e., *PKD1-GLA*-mutated patients); cases in which the molecular results have had a decisive impact on management and correct therapeutic decisions.

Therefore, genetic analysis can help to reach a timely diagnosis, which is extremely important as it can put an end to diagnostic uncertainty and allow for appropriate disease-specific counseling and the implementation of personalized medical plans in accordance with the current disease-specific consensus guidelines and, in the longer term, it can support the development of better designed clinical trials [59].

Future clinical studies including larger cohorts of patients, the identification of additional genes, follow-up information and comparative analysis of healthy controls are needed. It is worth noting that in the *PKD1* gene, the presence of six pseudogenes is a complicating issue in NGS, because their sequence can interfere with the detection of canonical variants and generate false-positive and false-negative variants; here, we have tried to overcome this issue using specific primers designed to exclude DNA regions belonging to the six pseudogenes [46]. Additional technologies such as Whole-Exome Sequencing (WES) or Whole-Genome Sequencing (WGS) could provide an alternative type of analysis or a second-step analysis of those patients in whom the mutation cannot be identified through NGS analysis. However, it should be noted that there are some limitations which make these approaches not easily feasible, such as the much longer turnaround times of WES and WGS to complete the diagnostic process together with the very high cost that do not always make the choice of these technological platforms appropriate. Molecular analysis will help to confirm the diagnosis in clinically uncertain/atypical cases, exclude the presence of a variant in donors for kidney transplantation for living donors, and offer genetic counselling for at-risk families [16,23,31,46,47].

## 5. Conclusions

In conclusion, our study confirms the high degree of genetic heterogeneity within the Italian population, further endorsing that several pathogenic mutations in different genes can be the molecular basis of the same clinical phenotype of PKD patients. An accurate and prompt but more importantly early molecular diagnosis is essential to adopt timely lifestyle choices and might also inform reproductive counselling, and could also be interesting as a predictive factor for family members. Currently, the greater chances of positive PKD clinical trial outcomes and lower drug dropouts are closely linked to a more precise and specific molecular diagnosis, highlighting the key role of molecular characterization in PKDs. Finally, molecular analysis helped to exclude the presence of a variant in donors for kidney transplantation and to offer genetic counselling for at-risk families.

## Figures and Tables

**Table 1:** Genetic classification of PKD population.

		ADPKD Patients (*n*)	Age at Clinical Diagnosis (Mean ± SD)	Sex (%)	Family History (%)	eGFR (Mean ± SD)
	***PKD1***	124	26.57 ± 12.97	M (43.9)	73.17	73.54 ± 33.54
	***PKD2***	31	39.6 ± 11.68		64.5	54.1 ± 33.29
	***GANAB***	4	20.12 ± 6.2	M (25)	100	79.25 ± 13.48
	***HNF1B***	1	0	F	No	83
	***VHL***	2	26 ± 12.72	M (50)	100	100
	***UMOD***	2	38 ± 9.89	F (100)	100	39.5 ± 27.57
	***TSC2***	1	11	F	No	90
	***LRP5***	2	17.5 ± 17.6	F	100	46.3 ± 13.91
**Genes bearing LP/P variants**		**ARPKD Patients (*n*)**	**Age at clinical diagnosis (mean ± SD)**	**Sex (%)**	**Family** **history (%)**	**eGFR (mean ± SD)**
	***PKHD1***	2	25 ± 4.94	F (100)	0	75 ± 11.31
	***MAPKBP1***	1	20	M	No	9
		**Trans-heterozygous patients**	**Age at clinical diagnosis**	**Sex**	**Family** **history**	**eGFR (mean± SD)**
	***CRB2***	1	58	F	Yes	100
	***PMM2***					
	***CC2D2A***	1	45	F	Yes	48
	***PMM2***					
		**Patients with singleton status (*n*)**	**Age at clinical diagnosis (mean ± SD)**	**Sex (%)**	**Family** **history (%)**	**eGFR (mean ± SD)**
	***PMM2***	2	60 ± 21.21	M (50)	100	35.5 ± 17.6
	***CEP164***	1	26	M	No	71
	***ATP6V1B1***	1	58	M	No	60
**Genes bearing VUS variants**		**ADPKD Patients (*n*)**	**Age at clinical diagnosis (mean ± SD)**	**Sex (%)**	**Family** **history (%)**	**eGFR (mean ± SD)**
	***PKD1***	18	33.09 ± 20,39	M (61.5)	Yes (77.7)	74.21 ± 35.47
	***PKD2***	2	45 ±4.24	M (50)	Yes (100)	57.5 ± 20.50
	***LRP5***	2	61 ± 8.48	F (100)	Yes (50)	64.5 ± 37.47
	***HNF1B***	1	66	M	Yes	37
	***ACTN4***	1	49	M	Yes	81
		**ARPKD Patients (*n*)**	**Age at clinical diagnosis (mean ± SD)**	**Sex (%)**	**Family** **history (%)**	**eGFR (mean ± SD)**
	***PKHD1***	2	35.5 ± 45.96	M (100)	Yes (100)	74 ± 8.48
		**Trans-heterozygous patients**	**Age at clinical diagnosis (mean ± SD)**	**Sex**	**Family** **history**	**eGFR (mean ± SD)**
	***FOXI1*** ***TTC21B***	1	birth	F	Yes	78
	***COL4A4*** ***INVS***	1	46	F	Yes	43
	***PKHD1 CD2AP***	1	55	F	Yes	72
	***NPHS2*** ***ANKS6***	1	75	F	No	43
	***FOXI1 TTC21b***	1	63	M	No	80
	***BBS4*** ***NPHS1***	1	68	M	No	39
		**Patients with singleton status (*n*)**	**Age at clinical diagnosis (mean ± SD)**	**Sex (%)**	**Family** **history (%)**	**eGFR (mean ± SD)**
	***PKHD1***	3	57 ± 13.85	M (66.6)	Yes (66.6)	67.33 ± 2.08
	***NPHS1***	2	55 ± 15.91	M (100)	Yes (100)	73 ± 20.2
	***NPHS2***	1	69	M	Yes	78
	***NPHS3***	1	74	M	No	67
	***CEP290***	1	4	M	No	20
	***MAPKBP1***	1	47	M	Yes	22
	***CD2AP***	1	50	M	Yes	80
	***MYO1E***	1	27	F	Yes	33
	***ZNF423***	1	48	F	Yes	93
	***CC2D2A***	1	61	M	Yes	60
	***NEK8***	2	61.5 ± 10.8	M (100)	Yes (100)	68.89 ± 20.12
	**No mutation**	32	52.6 ± 19.74	M (56)	Yes (56.7)	58.68 ± 37.02
	**Total patients**	255				

**Table 2:** Genotype description of ARPKD recessive patients (compound heterozygous).

Gene	c.DNA Change	Region	Protein Variant	ACMG Classification and Sub-Criteria	gnomAD Allele Frequency	Coding Impact	Age at Diagnosis	Liver Cysts	e-GFR	CKD-Stage	Literature and/or ClinVar Reference
***PKHD1***	c.1690C>T	Exon 18	p.Arg564Ter	VUS	0.0000239	Missense	32	Yes	67	G2	VCV000594333.13—ClinVar—NCBI (nih.gov)
	c.9107T>G	Exon 58	p.Val3036Gly			Missense					
	c.431C>T	Exon 6	p.Pro144Leu	Pathogenic (PP3-PM2-PP5-BP1)	0.0000239	Missense	22	No	83	G2	VCV000594333.13—ClinVar—NCBI (nih.gov)
	c.9107T>G	Exon 58	p.Val3036Gly			Missense					
	c.10036T>C	Exon 60	p.Cys3346Arg	VUS	Not found	Missense	68	Yes	68	G2	-
	c.5585C>T	Exon 34	p.Ser1862Leu	Pathogenic (PP3-PM2-PP5-BP1)		Missense					
	c.5665G>A	Exon 35	p.Glu1889Lys	VUS	Not found	Missense	1	No	82	G2	-
	c.7911+19T>C	Int49	-	VUS		Intronic					
***MAPKBP1***	c.2271delA	Exon 19	p.Gly758AspfsTer74	VUS	Not found	Nonsense	46	No	9	G5	-
				VUS						(age at ESKD: 46)	

**Table 3:** Genotype description of PKD patients with two variants in different recessive genes (trans-heterozygous patients).

Gene	c.DNA Change	Region	Protein Variant	ACMG Classification and Sub-Criteria	gnomAD Allele Frequency	Coding Impact	Age at Diagnosis	Liver Cysts	e-GFR	CKD-Stage	Literature and/or ClinVar Reference
***CRB2***	c.357T>G	Exon 2	p.His119Gln	VUS	Not found	Missense	58	No	100	Normal	VCV000198714.31—ClinVar—NCBI (nih.gov)
***PMM2***	c.713G>A	Exon 8	p.Arg238His	Likely Pathogenic (PM1-PM5-PM2-PP5-BP4)		Missense					
***FOXI1***	c.908G>A	Exon 2	p.Gly303Glu	VUS	Not found	Missense	At birth	No	78	G2	-
***TTC21b***	c.667C>G	Exon 6	p.Leu223Val	VUS		Missense					
***COL4A4***	c.1460G>A	Exon 22	p.Gly487Glu	VUS	Not found	Missense	46	Yes	43	G3b	-
***INVS***	c.2296C>A	Exon 15	p.Leu766Met	VUS		Missense					
***NPHS2***	c.686G>A	Exon 5	p.Arg229Gln	VUS	Not found	Missense	75	No	43	G3b	-
***ANKS6***	c.1400G>A	Exon 7	p.Arg467Gln	VUS		Missense					
***FOXI1***	c.908G>A	Exon 2	p.Gly303Glu	VUS	Not found	Missense	63	No	70	Normal	-
***TTC21b***	c.667C>G	Exon 6	p.Leu223Val	VUS		Missense					
***BBS4***	c.1027C>A	Exon 12	p.Leu343Ile	VUS	Not found	Missense	62	No	75	Normal	-
***NPHS1***	c.2819G>T	Exon 21	p.Arg940Leu	VUS		Missense					
***CC2D2A***	c.823a>g	Exon 10	p.Ile275Val	VUS	Not found	Missense	46	No	48	G3b	[22]
***PMM2***	c.422g>a	Exon 5	p.Arg141His	Pathogenic (PS3-PP5-PM1-PM5-PM2-BP4)		Missense					
***PKHD1***	c.11338C>T	Exon 63	p.Pro3780Ser	VUS	Not found	Missense	52	Yes	40	G3b	-
***CD2AP***	c.491A>T	Exon 5	p.Glu164Val	VUS		Missense					

**Table 4:** Genotype description of truncating mutations.

	cDNA Change	Region	Protein Variant	ACMG Classification and Sub-Criteria	gnomAD Allele Frequency	Literature and/or ClinVar Reference
***PKD1***	c.12008dup	Exon 44	p.Gln4005AlafsTer152	Likely Pathogenic PVS1-PM2)	Not found	-
	c.12058C>T	Exon 44	p.Arg4021Ter	Pathogenic (PVS1-PP5-PM2)	Not found	[23]
	c.11705_11708del	Exon 42	p.Thr3902ArgfsTer41	Likely Pathogenic (PVS1-PM2)	Not found	-
	c.8698C>T	Exon 23	p.Gln2900Ter	Pathogenic (PVS1-PP5-PM2)	Not found	VCV000562271.2— ClinVar—NCBI (nih.gov)
	c.7984C>T	Exon 21	p.Gln2662Ter	Pathogenic (PVS1-PP5-PM2)	0.000004405	VCV000636940.5— ClinVar—NCBI (nih.gov)
	c.12908A>T	Exon 46	p.4303LeuextTer35	Likely Pathogenic (PM4-PM2-BP4)	Not found	-
	c.3745delG	Exon 15	p.Asp1249ThrfsTer24	Pathogenic (PVS1-PP5-PM2)	Not found	VCV000972837.1— ClinVar—NCBI (nih.gov)
	c.11438_11439del	Exon 41	p.Tyr3813Ter	Likely Pathogenic (PVS1-PM2)	Not found	-
	c.11881C>T	Exon 43	p.Gln3961Ter	Pathogenic (PVS1-PP5-PM2	Not found	VCV000997175.4— ClinVar—NCBI (nih.gov)
	c.5869_5870dup	Exon 15	p.Ser1957ArgfsTer16	Likely Pathogenic (PVS1-PM2)	Not found	-
	c.3398_3399delTG	Exon 15	p.Val1133GlufsTer2	Pathogenic (PVS1-PP5-PM2)	Not found	[16]
	c.8238del	Exon 23	p.Met2747TrpfsTer9	Likely Pathogenic (PVS1-PM2)	Not found	-
	c.4951C>T	Exon 15	p.Gln1651Ter	Pathogenic (PVS1-PP5-PM2	Not found	PMID: 23431742
	c.1198C>T	Exon 5	p.Arg400Ter	Pathogenic (PVS1-PP5-PM2)	0.00001398	[24]
	c.11568C>G	Exon 42	p.Tyr3856Ter	Likely Pathogenic (PVS1-PM2)	Not found	-
	c.3706C>T	Exon 15	p.Gln1236Ter	Pathogenic (PVS1-PP5-PM2)	Not found	[16]
	c.11571C>G	Exon 42	p.Tyr3857Ter	Likely Pathogenic (PP3-PM1-PM2-BP1)	Not found	-
	c.10591C>T	Exon 35	p.Gln3531Ter	Likely Pathogenic (PVS1-PM2)	Not found	-
	c.10459C>T	Exon 34	p.Gln3487Ter	Pathogenic (PVS1-PP5-PM2)	0	[23]
	c.4888C>T	Exon 15	p.Gln1630Ter	Pathogenic ((PVS1-PP5-PM2)	Not found	[25]
	c.5154_5163dup	Exon 15	p.Met1722GlyfsTer52	Likely Pathogenic (PVS1-PM2)	Not found	-
	c.7597_7598del	Exon 19	p.Ser2533GlnfsTer61	Pathogenic (PVS1-PP5-PM2)	Not found	[26]
	c.11967_11974dup	Exon 43	p.Ser3992TrpfsTer49	Likely Pathogenic (PVS1-PM2)	Not found	-
	c.8371_8372dup	Exon 23	p.Ser2792GlyfsTer84	Likely Pathogenic (PVS1-PM2)	Not found	-
	c.2215dup	Exon 11	p.Gln739ProfsTer59	Likely Pathogenic (PVS1-PM2)	Not found	-
	c.5014_5015delAG	Exon 15	p.Arg1672GlyfsTer98	Pathogenic (PVS1-PP5-PM2)	Not found	[27]
	c.10722G>A	Exon 7	p.Trp3574Ter	Pathogenic (PVS1-PP5-PM2…)	Not found	VCV000997310.4— ClinVar—NCBI (nih.gov)
	c.11646_11659del	Exon 42	p.Ser3883CysfsTer72	Pathogenic (PVS1-PP5-PM2)	Not found	VCV000811475.8— ClinVar—NCBI (nih.gov)
	c.11560_11561del	Exon 42	p.Thr3854AlafsTer105	Pathogenic (PVS1-PP5-PM2)	Not found	VCV000522397.6— ClinVar—NCBI (nih.gov)
	c.7416_7417ins	Exon 18	p.Gly2473ArgfsTer28	Likely Pathogenic (PVS1-PM2)	Not found	-
	c.5607dupC	Exon 15	p.Asn1870GlnfsTer120	Likely Pathogenic (PVS1-PM2)	Not found	-
	c.2085delC	Exon 10	p.Ala696ArgfsTer89	Pathogenic (PVS1-PP5-PM2)	Not found	VCV000811793.10— ClinVar—NCBI (nih.gov)
	c.1105_1106delAG	Exon 5	p.Ser369Ter	Pathogenic (PVS1-PM2)	Not found	-
	c.10420C>T	Exon 34	p.Gln3474Ter	Likely Pathogenic (PVS1-PM2)	Not found	-
	c.3520_3527del	Exon 15	p.Gln1174Cysfs34Ter	Likely Pathogenic (PVS1-PM2)	Not found	-
	c.3802C>T	Exon 15	p.Gln1268Ter	Likely Pathogenic (PVS1-PM2)	Not found	-
	c.6199C>T	Exon 15	p.Gln2067Ter	Pathogenic (PVS1-PP5-PM2)	Not found	-
	c.9425_9426ins	Exon 27	p.Tyr3143ValfsTer36	Likely Pathogenic (PVS1-PM2)	Not found	-
	c.1987C>T	Exon 10	p.Gln663Ter	Pathogenic (PVS1-PP5-PM2)	Not found	[28]
	c.3349C>T	Exon 15	p.Gln1117Ter	Pathogenic (PVS1-PP5-PM2)	Not found	[29]
	c.11267-1G>T	Int39	-	Likely Pathogenic (PVS1-PM2)	Not found	-
	c.9771_9774delCTTT	Exon 29	p.Phe3257LeufsTer58	Likely Pathogenic (PVS1-PM2)	Not found	-
	c.2711_2712delAG	Exon 11	p.Glu904GlyfsTer196	Likely Pathogenic (PVS1-PM2)	Not found	-
	c.3067C>T	Exon 13	p.Gln1023Ter	Pathogenic (PVS1-PP5-PM2)	Not found	[30]
	c.427C>T	Exon 4	p.Gln143Ter	Likely Pathogenic (PVS1-PM2)	Not found	-
	c.11357_11361dup	Exon 40	p.His3788ValfsTer39	Likely Pathogenic (PVS1-PM2)	Not found	-
	c.10549G>T	Exon 35	p.Glu3517Ter	Likely Pathogenic (PVS1-PM2)	Not found	-
	c.6560G>A	Exon 15	p.Trp2187Ter	Pathogenic (PVS1-PP5-PM2)	0	VCV000997397.1— ClinVar—NCBI (nih.gov)
	c.11763G>A	Exon 43	p.Trp3922Ter	Pathogenic (PVS1-PP5-PM2)	Not found	VCV000997191.2— ClinVar—NCBI (nih.gov)
	c.3202C>T	Exon 14	p.Gln1068Ter	Pathogenic (PVS1-PP5-PM2)	Not found	VCV000972875.4— ClinVar—NCBI (nih.gov)
	c.271_272delTC	Exon 2	p.Ser91GlyfsTer22	Likely Pathogenic (PVS1-PM2)	Not found	-
	c.2028dupC	Exon 10	p.Gly677ArgfsTer37	Likely Pathogenic (PVS1-PM2)	Not found	-
	c.12010C>T	Exon 44	p.Gln4004Ter	Likely Pathogenic (PVS1-PM2)	0.000004091	-
	c.5905G>T	Exon 15	p.Glu1969Ter	Likely Pathogenic (PVS1-PM2)	Not found	-
	c.6504C>G	Exon 15	p.Tyr2168Ter	Likely Pathogenic (PVS1-PM2)	Not found	-
	c.10894_10895del	Exon 37	p.Ser3632ProfsTer88	Likely Pathogenic (PVS1-PM2)	Not found	-
	c.5884C>T	Exon 15	p.Gln1962Ter	Pathogenic (PVS1-PP5-PM2)	Not found	VCV000916425.13— ClinVar—NCBI (nih.gov)
	c.3514C>T	Exon 14	p.Gln1172Ter	Likely Pathogenic (PVS1-PM2)	Not found	-
	c.1837C>T	Exon 8	p.Gln613Ter	Pathogenic (PVS1-PP5-PM2)	Not found	[31]
	c.2117delA	Exon 10	p.Lys706ArgfsTer10	Likely Pathogenic (PVS1-PM2)	Not found	-
	c.261G>A	Exon 1	p.Trp87Ter	Pathogenic (PVS1-PP5-PM2)	Not found	VCV000805202.1— ClinVar—NCBI (nih.gov)
	c.1249C>T	Exon 5	p.Arg417Ter	Pathogenic (PVS1-PP5-PM2)	Not found	[27]
	c.2419C>T	Exon 13	p.Arg807Ter	Pathogenic (PVS1-PP5-PM2)	Not found	[32]
	c.958C>T	Exon 4	p.Arg320Ter	Pathogenic (PVS1-PP5-PM2)	Not found	[10]
	c.1395T>A	Exon 6	p.Tyr465Ter	Likely Pathogenic (PVS1-PM2)	0.000003982	-
	c.2358del	Exon 10	p.Glu787ArgfsTer14	Pathogenic (PVS1-PP5-PM2)	Not found	-
	c.916C>T	Exon 4	p.Arg306Ter	Pathogenic (PVS1-PP5-PM2)	0.000003981	[24]
	c.637C>T	Exon 2	p.Arg213Ter	Pathogenic (PVS1-PP5-PM2)	0.00003188	[33]
	c.2614C>T	Exon 14	p.Arg872Ter	Pathogenic (PVS1-PP5-PM2)	0.000003983	-
***VHL***	c.217C>T	Exon 1	p.Gln73Ter	Pathogenic (PVS1-PP5-PM2)	Not found	[34]
***PKHD1***	c.1690C>T	Exon 18	p.Arg564Ter	Pathogenic (PVS1-PP5-PM2)	0.00000398	[35]
***MAPKBP1***	c.2271delA	Exon 19	p.Gly758AspfsTer74	Likely Pathogenic (PVS1-PM2)	Not found	-

**Table 5:** Genotype description of patients bearing novel pathogenic/likely pathogenic variants.

Gene	cDNA Change	Region	Protein Variant	ACMG Classification and Criteria	Variant Type	Molecular Impact
***PKD1***	c.12008dup	Exon 44	p.Gln4005AlafsTer152	Likely Pathogenic PVS1-PM2)	Duplication	Frameshift
	c.11705_11708del	Exon 42	p.Thr3902ArgfsTer41	Likely Pathogenic (PVS1-PM2)	Deletion	Frameshift
	c.5869_5870dup	Exon 15	p.Ser1957ArgfsTer16	Likely Pathogenic (PVS1-PM2)	Duplication	Frameshift
	c.8238del	Exon 23	p.Met2747TrpfsTer9	Likely Pathogenic (PVS1-PM2)	Deletion	Frameshift
	c.5154_5163dup	Exon 15	p.Met1722GlyfsTer52	Likely Pathogenic (PVS1-PM2)	Duplication	Frameshift
	c.7597_7598del	Exon 19	p.Ser2533GlnfsTer61	Pathogenic (PVS1-PP5-PM2)	Deletion	Frameshift
	c.11967_11974dup	Exon 43	p.Ser3992TrpfsTer49	Likely Pathogenic (PVS1-PM2)	Duplication	Frameshift
	c.8371_8372dup	Exon 23	p.Ser2792GlyfsTer84	Likely Pathogenic (PVS1-PM2)	Duplication	Frameshift
	c.2215dup	Exon 11	p.Gln739ProfsTer59	Likely Pathogenic (PVS1-PM2)	Duplication	Frameshift
	c.7416_7417ins	Exon 18	p.Gly2473ArgfsTer28	Likely Pathogenic (PVS1-PM2)	Insertion	Frameshift
	c.5607dup	Exon 15	p.Asn1870GlnfsTer120	Likely Pathogenic (PVS1-PM2)	Duplication	Frameshift
	c.3520_3527del	Exon 15	p.Gln1174Cysfs34Ter	Likely Pathogenic (PVS1-PM2)	Deletion	Frameshift
	c.9425_9426ins	Exon 27	p.Tyr3143ValfsTer36	Likely Pathogenic (PVS1-PM2)	Insertion	Frameshift
	c.11357_11361dup	Exon 40	p.His3788ValfsTer39	Likely Pathogenic (PVS1-PM2)	Duplication	Frameshift
	c.7864_7899del	Exon 21	p.Tyr2622_Lys2633del	Likely Pathogenic (PS2-PM4-PM2)	Deletion	In frame
	c.10973_10987del	Exon 37	p.Glu3658_Lys3662del	Likely Pathogenic (PM1-PM4-PM2)	Deletion	In frame
	c.10807G>C	Exon 36	p.Glu3603Gln	Likely Pathogenic (PM1-PM5-PM2-PP3)	SNV	Missense
	c.5609A>G	Exon 15	p.Asn1870Ser	Pathogenic(PM5-PP3-PM1-PM2)	SNV	Missense
	c.11534G>T	Exon 41	p.Arg3845Met	Likely Pathogenic (PP3-PM2-PM1-BP1)	SNV	Missense
	c.11438_11439del	Exon 41	p.Tyr3813Ter	Likely Pathogenic (PVS1-PM2)	Deletion	Frameshift
	c.11568C>G	Exon 42	p.Tyr3856Ter	Likely Pathogenic (PVS1-PM2)	SNV	Nonsense
	c.10591C>T	Exon 35	p.Gln3531Ter	Likely Pathogenic(PVS1-PM2)	SNV	Nonsense
	c.3802C>T	Exon 15	p.Gln1268Ter	Likely Pathogenic (PVS1-PM2)	SNV	Nonsense
	c.427C>T	Exon 4	p.Gln143Ter	Likely Pathogenic (PVS1-PM2)	SNV	Nonsense
	c.3295+2T>C	Int14	-	Likely Pathogenic (PVS1-PM2)	SNV	Nonsense
	c.359+2T>G	Int3	-	Pathogenic (PVS1-PP5-PM2)	SNV	Splicing
	c.10217+2T>G	Int32	-	Likely Pathogenic (PVS1-PM2)	SNV	Splicing
	c.11267-1G>T	Int39	-	Likely Pathogenic (PVS1-PM2)	SNV	Splicing
	c.12908A>T	Exon 46	p.4303LeuextTer35	Likely Pathogenic (PM4-PM2-BP4)	SNV	Stoploss
	c.2028dupC	Exon 10	p.Gly677ArgfsTer37	Likely Pathogenic (PVS1-PM2)	Duplication	Frameshift
	c.10549G>T	Exon 46	p.Glu3517Ter	Likely Pathogenic (PVS1-PM2)	SNV	Nonsense
***PKD2***	c.1244T>G	Exon 5	p.Leu415Arg	Likely Pathogenic (PP3-PM1-PM2)	SNV	Missense
	c.1142G>T	Exon 5	p.Gly381Val	Likely Pathogenic (PM1-PP3-PM2-BP1)	SNV	Missense
	c.1395T>A	Exon 6	p.Tyr465Ter	Likely Pathogenic (PVS1-PM2)	SNV	Nonsense
	c.2358delG	Exon 10	p.Glu787ArgfsTer14	Pathogenic (PVS1-PP5-PM2)	Deletion	Splicing
***MAPKBP1***	c.2271delA	Exon 19	p.Gly758AspfsTer74	Likely Pathogenic (PVS1-PM2)	SNV	Frameshift
***UMOD***	c.767G>A	Exon 3	p.Cys256Tyr	Likely Pathogenic (PP3-PM1-PM2)	SNV	Missense

## Data Availability

Not applicable.

## Supplementary Materials

The following supporting information can be downloaded at https://www.mdpi.com/article/10.3390/genes14061236/s1, Table S1: Genes comprised in the PKD disease NGS panel; Table S2: Pathogenic and likely pathogenic mutations in AD genes; Table S3: Pathogenic and likely pathogenic mutations in AR genes; Table S4: VUS variants in AD genes; Table S5: VUS alterations in AR genes.
