# Supplementary material for: Molecular Diagnosis and Identification of Novel Pathogenic Variants in a Large Cohort of Italian Patients Affected by Polycystic Kidney Diseases
Source: Genes (Basel). 2023 Jun 8;14(6):1236. doi: 10.3390/genes14061236 (PMC10298188; doi:10.3390/genes14061236)
Supplement: Supplementary file 1 [file genes-14-01236-s001.zip › Supplementary Table S1.pdf]

Supplementary Table S1: Genes comprised in the PKD disease NGS panel.

	Gene symbol	Official Full Name	Function	Mode of inheritance	Phenotypes
1	ACTN4	Actinin, alpha-4	ACTN4 may act to anchor microfilaments to various cellular structures. Alpha-actinin is highly expressed in the glomerular podocyte, and is upregulated early in the course of some animal models of nephrotic syndrome.	autosomal dominant	Focal segmental glomerulosclerosis
2	AHI1	Abelson helper integration site 1	AHI1 is a component of a protein complex in the basal body, a ring-like structure that functions in the transition zone at the base of cilia. This complex acts as a barrier to restrict protein diffusion between plasma and ciliary membranes.	autosomal recessive	Joubert syndrome 3
3	ANLN	Actin-binding protein anillin	ANLN is an actin-binding protein that functions in cell growth and migration, as well as in cytokinesis.	autosomal dominant	Focal segmental glomerulosclerosis
4	ANKS6	Ankyrin repeat and sterile alpha motif domains-containing protein 6	ANKS6 assembles a complex of ciliary proteins required for renal and cardiovascular development.	autosomal recessive	Nephronophthisis 16 - Polycystic kidney disease - Heterotaxy syndrome
5	ATP6V0A4	ATPase, H+ transporting, lysosomal, v0 subunit a, isoform 4	Vacuolar-type proton pumps help maintain acid-base homeostasis either within intracellular compartments or at plasma membranes. The pumps are made up of 13 subunits, which form 2 functional domains: a V1 domain where ATP hydrolysis provides energy for proton movement, and a membrane-anchored V0 domain where proton translocation takes place. ATP6V0A4 is 1 of the subunits that form the V0 domain.	autosomal recessive	Distal renal tubular acidosis 3, with or without sensorineural hearing loss
6	ATP6V1B1	ATPase, H+ transporting, lysosomal, 56/58-kd, v1 subunit b, isoform 1	ATP6B1 encodes a component of vacuolar ATPase (V-ATPase), a multisubunit enzyme that mediates acidification of intracellular organelles. V-ATPases pump protons against an electrochemical gradient.	autosomal recessive	Distal renal tubular acidosis 2 with progressive sensorineural hearing loss

7	BBS4	BBS4	BBS4 is 1 of 7 BBS proteins that form the stable core of a protein complex required for ciliogenesis.	autosomal recessive	Bardet-Biedl syndrome
8	BBS9	BBS9	BBS9 is an autosomal recessive disorder characterized by obesity, polydactyly, renal anomalies, retinopathy, and mental retardation.<br><br>For a general phenotypic description and a discussion of genetic heterogeneity of Bardet-Biedl syndrome, see BBS1.	autosomal recessive	Bardet-Biedl syndrome9
9	BBS10	BBS10	The BBS10 gene is likely to perform ciliary functions specific to vertebrates. The protein is thought to be involved in the regulation of WNT as well.	autosomal recessive	Bardet-Biedl syndrome10
10	BBS12	BBS12 Gene	The BBS12 gene is likely to perform ciliary functions specific to vertebrates The protein is thought to be involved in the regulation of WNT as well.	autosomal recessive	Bardet-Biedl syndrome12
11	CA2	Carbonic Anhydrase II	Carbonic anhydrase II (CA) is a member of a family of zinc metalloenzymes.	autosomal recessive	Osteopetrosis, autosomal recessive 3, with renal tubular acidosis
12	CC2D2A	Coiled-coil and c2 domains-containing protein 2A	CC2D2A is a component of a protein complex in the basal body that functions in the transition zone at the base of cilia. This complex acts as a barrier to restrict protein diffusion between plasma and ciliary membranes.	autosomal recessive	Joubert syndrome 9 - Meckel syndrome 6 - Joubert syndrome
13	CD2AP	CD2-associated protein	CD2AP is a critical scaffolding protein that facilitates intercellular junctions.		Focal segmental glomerulosclerosis
14	CEP83	Centrosomal protein 83-KD	CEP83 is required for the docking of ciliary vesicles derived from the Golgi complex to the mother centriole, the interaction which initiates primary cilium assembly.	autosomal recessive	Nephronophthisis 18
15	CEP164	Centrosomal protein 164-KD	CEP164 is a basal body protein required for assembly of the primary cilium. CEP164 also has a critical role in DNA damage response.	autosomal recessive	Nephronophthisis 15 - Primary ciliary dyskinesia

16	CEP290	Centrosomal protein 290-KD	The CEP290 gene encodes a centrosomal protein involved in ciliary assembly and ciliary trafficking.	autosomal recessive	Meckel syndrome 4 - Bardet-Biedl syndrome - Joubert syndrome - Meckel syndrome Nephronophthisis - Skeletal dysplasia - Leber congenital amaurosis
17	COL4A3	Collagen, type IV, lpha-3	Type IV collagen is found only in basement membranes, where it is the major structural component. COL4A3 is 1 of 6 alpha chains that form the heterotrimeric type IV collagen molecules.	autosomal dominant / autosomal recessive	Alport syndrome 3, Alport syndrome 2
18	COL4A4	Collagen, type IV, alpha-4	Type IV collagen is found only in basement membranes, where it is the major structural component. COL4A4 is 1 of 6 alpha chains that form the heterotrimeric type IV collagen molecules.	autosomal recessive	Alport syndrome 2
19	COL4A5	Collagen, type IV, alpha-5	Using a zebrafish model, it has been shown that Col4a5 on the surface of the tectum basement membrane bound Slit1 and guided retinal ganglion cell axons expressing.	X-linked	Alport syndrome 1
20	CRB2	Crumbs cell polarity complex component 2	The CRB2 gene encodes a polarity complex protein	autosomal recessive	Focal segmental glomerulosclerosis 9
21	DCDC2	Doublecortin domain-containing protein 2	DCDC2 plays a role in the inhibition of canonical WNT signaling. DCDC2 is a ciliary protein that binds tubulin and enhances microtubule polymerization.	autosomal recessive	Nephronophthisis 19
22	FOXI1	Forkhead box I1	Fkhl1 is a transcription factor which, if mutated, can cause the onset of renal tubular acidosis Distal.	autosomal recessive	Renal tubular acidosis Distal
23	GLIS2	Glis family zinc finger protein 2	GLIS2 is a zinc finger protein implicated in transcriptional regulation.		Nephronophthisis 7
24	GREB1L	Greb1-like retinoic acid receptor coactivator	GREB1L is predicted to be involved in retinoic acid signaling.	autosomal dominant	Renal hypodysplasia/aplasia 3

25	INVS	Inversion of embryonic turning; inv<br>nephrocystin 2	INVS is part of a complex of ciliary proteins required for renal and cardiovascular development.	autosomal recessive	Polycystic kidney disease - Nephronophthisis 2
26	LRP6	Low density lipoprotein receptor-related protein 6	he LRP6 gene encodes a member of the low density lipoprotein receptor (LDLR) gene family, which consists of cell surface proteins involved in receptor-mediated endocytosis of specific ligands. LRP6 and LRP5 play a role in WNT signaling.	autosomal dominant	Kidney diseases
27	MAPKBP1	Mitogen-activated protein kinase-binding protein 1	Mitogen-activated protein kinases (MAPKs) form a kinase tier module in which MAPK, MAPKK, and MAPKKK are held by scaffold proteins. The scaffold proteins, such as MAPKBP1, serve as a protein platform for selective and spatial kinase activation.	autosomal recessive	Nephronophthisis 20
28	MYO1E	MYOSIN IE	The MYO1E gene encodes a nonmuscle membrane-associated class I myosin with a motor-head domain that binds ATP and F-actin, a calmodulin-binding neck domain, and a tail domain. MYO1E is expressed in the podocyte membrane in the renal glomerulus.	autosomal recessive	Focal segmental glomerulosclerosis
29	NPHS1	Nephrin	The NPHS1 gene encodes nephrin, a kidney glomerular filtration barrier protein that is an essential component of the interpodocyte-spanning slit diaphragm.	autosomal dominant	Nephrotic syndrome, type 1 - Focal segmental glomerulosclerosis
30	NPHS2	Podocin	The NPHS2 gene encodes podocin, a protein that is expressed at the insertion of the slit diaphragm in the renal glomerulus. Podocin is thus crucial in the establishment of the glomerular filtration barrier.	autosomal recessive	Nephrotic syndrome, type 2 -Focal segmental glomerulosclerosis
31	NEK8	Nima-related Kinase 8	NEK8 is part of a complex of ciliary proteins required for renal and cardiovascular development. NEK8 is also critical for cell cycle regulation through the Hippo signaling pathway.	autosomal recessive	Nephronophthisis 9/Renal-hepatic-pancreatic dysplasia 2 -
32	NOTCH2	Notch receptor 2	The NOTCH2 gene encodes a single pass transmembrane protein belonging to an evolutionarily conserved NOTCH receptor family. NOTCH signaling is activated through cell-cell contact: it regulates gene expression in association with transcriptional cofactors.	autosomal dominant	Polycystic kidney disease - Primary ciliary dyskinesia - Skeletal dysplasia - Hajdu-Cheney syndrome - Alagille syndrome 2

33	NPHP3	Nephrocystin 3	NPHP3 is part of a complex of ciliary proteins required for renal and cardiovascular development.	autosomal recessive	Polycystic kidney disease - Meckel syndrome Nephronophthisis - Renal-hepatic-pancreatic dysplasia 1/Nephronophthisis 3/Meckel syndrome 7
34	PAX2	Paired box gene 2	In the developing kidney, induction of nephrogenesis by the ureter is accompanied by an increase in expression levels of the PAX2 gene.	autosomal dominant	Focal segmental glomerulosclerosis - Glomerulosclerosis, focal segmental, 7
35	PMM2	Phosphomannomutase 2	The PMM2 gene encodes phosphomannomutase, an enzyme necessary for the synthesis of GDP-mannose.	autosomal recessive	Congenital disorder of glycosylation, type Ia
36	SEC61A	SEC61 translocon, alpha-1 subunit	SEC61A1 is a subunit of the heteromeric SEC61 complex, which also contains beta and gamma subunits. The SEC61 complex forms the core of the mammalian endoplasmic reticulum (ER) translocon, a transmembrane channel for the translocation of proteins across the ER membrane.	autosomal dominant	Tubulointerstitial kidney disease, autosomal dominant, 5 - Familial juvenile hyperuricemic nephrophaty type 4
37	SLC4A1	Solute carrier family 4 (anion exchanger), member 1	Band 3 is the major glycoprotein of the erythrocyte membrane. It mediates exchange of chloride and bicarbonate across the phospholipid bilayer and plays a central role in respiration of carbon dioxide.	autosomal dominant	Distal renal tubular acidosis 1 - Distal renal tubular acidosis 4 with hemolytic anemia
38	SLC4A4	Solute carrier family 4 (sodium bicarbonate cotransporter), member 4	Sodium bicarbonate cotransporters (NBCs) mediate the coupled movement of sodium and bicarbonate ions across the plasma membrane of many cells. This is an electrogenic process with an apparent stoichiometry of 3 bicarbonate ions per sodium ion. Sodium bicarbonate cotransport is involved in bicarbonate secretion/absorption and intracellular pH regulation.	autosomal recessive	Renal tubular acidosis, proximal, with ocular abnormalities
39	VHL	Von Hippel-Lindau tumor suppressor	The protein products of the VHL gene play a role in the oxygen-sensing pathway, in microtubule stability and orientation, tumor suppression, cilia formation, regulation of senescence, cytokine signaling, collagen IV regulation, and assembly of a normal extracellular fibronectin matrix.	autosomal dominant	von Hippel-Lindau syndrome
40	TTC21B	Tetratricopeptide repeat domain-containing protein 21b	The TTC21B gene encodes an axonemal protein required for retrograde intraflagellar transport and involved in ciliary function.	autosomal dominant / autosomal recessive	Skeletal ciliopathy - Joubert syndrome - Nephronophthisis 12

41	TRPC6	Transient receptor potential cation channel, subfamily c, member 6	TRPC6 is a nonselective cation channel that is activated by diacylglycerol (DAG) in a membrane-delimited fashion, independently of protein kinase C.	autosomal dominant	Focal segmental glomerulosclerosis
42	TMEM67	Proteina transmembrana 67, MKS3	MKS1 and MKS3 have roles in ciliogenesis and renal tubulogenesis.	autosomal recessive	Nephronophthisis11
43	WDR19	WD repeat-containing protein 19	The nephronophthisis-13 (NPHP13) is caused by homozygous or compound heterozygous mutation in the WDR19 gene (608151) on chromosome 4p14.	autosomal recessive	Skeletal ciliopathy - Nephronophthisis 13
44	ZNF423	Zinc finger protein 423	ZNF423 localizes to the ciliary transition zone and is also involved in DNA damage repair.	autosomal dominant / autosomal recessive	Joubert syndrome 19 - Nephronophthisis 14
45	ALG8	ALG8 alpha-1,3-glucosyltransferase	Required for PKD1/Polycystin-1 maturation and localization to the plasma membrane of the primary cilia. It adds the second glucose residue to the lipid-linked oligosaccharide precursor for N-linked glycosylation.	autosomal dominant	cystic liver disease; cystic kidney disease; Polycystic liver disease 3
46	ALG9	ALG9 alpha-1,2-mannosyltransferase	Catalyzes the transfer of mannose from Dol-P-Man to lipid-linked oligosaccharides.	monoallelic and autosomal recessive	cystic liver disease<br>cystic kidney disease<br>Gillessen-Kaesbach-Nishimura syndrome,
47	DNAJB11	DnaJ heat shock protein family (Hsp40) member B11	As a co-chaperone for HSPA5 it is required for proper folding, trafficking or degradation of proteins. It is necessary for maturation and correct trafficking of PKD1.	autosomal dominant	cystic kidney disease; end stage renal failure; Polycystic kidney disease; Tubulointerstitial kidney disease
48	DZIP1L	DAZ interacting zinc finger protein 1 like	Involved in primary cilium formation.	Autosomal recessive	ARPKD; Polycystic kidney disease 5

49	GLA	Galactosidase Alpha	Catalyzes the hydrolysis of glycosphingolipids and participates to their degradation in the lysosome.	autosomal dominant	Fabry disease, cardiomyopathy
50	GANAB	Glucosidase II alpha subunit	Required for PKD1/Polycystin-1 and PKD2/Polycystin-2 maturation and localization to the cell surface and cilia. Catalytic subunit of glucosidase II that cleaves sequentially the 2 innermost alpha-1,3-linked glucose residues from the oligosaccharide precursor of immature glycoproteins.	autosomal dominant	Mild cystic kidney and liver disease; Polycystic kidney disease 3
51	HNF1B	HNF1 homeobox B	Transcription factor, probably binds to the inverted palindrome 5'-GTTAATNATTAAC-3'.	autosomal dominant	Renal cysts and diabetes syndrome
52	LRP5	LDL receptor related protein 5	Acts as a coreceptor with members of the frizzled family of seven-transmembrane spanning receptors to transduce signal by Wnt proteins		Polycystic Liver Disease 4 With Or Without Kidney Cysts
53	OFD1	OFD1 centriole and centriolar satellite protein	Involved in the biogenesis of the cilium, a centriole-associated function.	X-linked	Oral-facial-digital syndrome 1; Joubert syndrome
54	PKD1	polycystin 1, transient receptor potential channel interacting	Component of a heteromeric calcium-permeable ion channel formed by PKD1 and PKD2 that is activated by interaction between PKD1 and a Wnt family member, such as WNT3A and WNT9B. Both PKD1 and PKD2 are required for channel activity. Involved in renal tubulogenesis.	autosomal dominant	Polycystic kidney disease, adult type I, Autosomal dominant polycystic kidney disease (ADPKD)
55	PKD2	polycystin 2, transient receptor potential cation channel	Component of a heteromeric calcium-permeable ion channel formed by PKD1 and PKD2 that is activated by interaction between PKD1 and a Wnt family member, such as WNT3A and WNT9B. Can also form a functional, homotetrameric ion channel. Functions as a cation channel involved in fluid-flow mechanosensation by the primary cilium in renal epithelium	autosomal dominant	Polycystic Kidney Disease, Autosomal Dominant<br><br>Polycystic kidney disease 2
56	PKHD1	PKHD1 ciliary IPT domain containing fibrocystin/polyductin	Promotes ciliogenesis in renal epithelial cells and therefore participates in the tubules formation and/ or ensures the maintenance of the architecture of the lumen of the kidney	autosomal recessive	Autosomal Recessive Polycystic Kidney Disease

57	PRKCSH	Protein kinase C substrate 80K-H	Regulatory subunit of glucosidase II that cleaves sequentially the 2 innermost alpha-1,3-linked glucose residues from the oligosaccharide precursor of immature glycoproteins. Required for efficient PKD1/Polycystin-1 biogenesis and trafficking to the plasma membrane of the primary cilia.	autosomal recessive	Polycystic Liver Disease
58	REN	Renin	Highly specific endopeptidase, whose only known function is to generate angiotensin I from angiotensinogen in the plasma, initiating a cascade of reactions that produce an elevation of blood pressure and increased sodium retention by the kidney.	autosomal dominant	Autosomal dominant tubulointerstitial kidney disease (ADTKD)
59	SEC61B	SEC61 translocon subunit beta	Component of SEC61 channel-forming translocon complex that mediates transport of signal peptide-containing precursor polypeptides across the endoplasmic reticulum. Required for PKD1/Polycystin-1 biogenesis		Polycistic liver disease; polycystic kidney disease 4
60	SEC63	SEC63 homolog, protein translocation regulator	Mediates cotranslational and post-translational transport of certain precursor polypeptides across endoplasmic reticulum	autosomal dominant	Polycystic liver disease; Unexplained kidney failure in young people; Renal ciliopathies. Cystic kidney disease; Rare multisystem ciliopathy disorders; Unexplained paediatric onset end-stage renal disease
61	TSC1	TSC complex subunit 1	In complex with TSC2, inhibits the nutrient-mediated or growth factor-stimulated phosphorylation of S6K1 and EIF4EBP1. Involved in microtubule-mediated protein transport	autosomal dominant	Tuberous Sclerosis; Kidney disease
62	TSC2	TSC complex subunit 2	In complex with TSC1, this tumor suppressor inhibits the nutrient-mediated or growth factor-stimulated phosphorylation of S6K1 and EIF4EBP1. May also play a role in microtubule-mediated protein transport	autosomal dominant	Focal Cortical Dysplasia, Type Ii; Tuberous Sclerosis
63	UMOD	Uromodulin	Functions in biogenesis and organization of the apical membrane of epithelial cells of the thick ascending limb of Henle's loop (TALH), where it promotes formation of complex filamentous gel-like structure that may play a role in the water barrier permeability	autosomal dominant	Medullary Cystic Kidney Disease 2<br><br>Hyperuricemic nephropathy, familial juvenile 1,
