# Supplementary material for: Molecular Diagnosis and Identification of Novel Pathogenic Variants in a Large Cohort of Italian Patients Affected by Polycystic Kidney Diseases
Source: Genes (Basel). 2023 Jun 8;14(6):1236. doi: 10.3390/genes14061236 (PMC10298188; doi:10.3390/genes14061236)
Supplement: Supplementary file 1 [file genes-14-01236-s001.zip › Supplementary Tables S2-S5.pdf]

Supplementary Table S2: Pathogenic and likely pathogenic mutations in AD genes

	cDNA change	Region	Protein variant	ACMG Classification	Coding impact
PKD1	c.10026delT	Ex30	p.Leu3343SerfsTer54	P	frameshift
	c.10217+2T>G	Int32	-	P	Splicing
	c.10420C>T	Ex34	p.Gln3474Ter	P	nonsense
	c.10459C>T	Ex34	p.Gln3487Ter	P	nonsense
	c.10549G>T	Ex35	p.Glu3517Ter	LP	nonsense
	c.10591C>T	Ex35	p.Gln3531Ter	P	nonsense
	c.10722G>A	Ex36	p.Trp3574Ter	P	nonsense
	c.10807G>C	Ex36	p.Glu3603Gln	LP	missense
	c.10894_10895del	Ex37	p.Ser3632ProfsTer88	P	frameshift
	c.10973_10987del	Ex37	p.Glu3658_Lys3662del	LP	in frame
	c.1105_1106del	Ex5	p.Ser369Ter	P	nonsense
	c.11267-1G>T	Int39	-	P	splicing
	c.11357_11361dup	Ex40	p.His3788Valfs*39	LP	frameshift
	c.1141G>A	Ex5	p.Gly381Ser	P	missense
	c.11438_11439del	Ex41	p.Tyr3813Ter	LP	nonsense
	c.11534G>T	Ex41	p.Arg3845Met	P	missense
	c.11560_11561del	Ex42	p.Thr3854AlafsTer105	P	frameshift
	c.11568C>G	Ex42	p.Tyr3856Ter	P	nonsense
	c.11571C>G	Ex42	p.Tyr3857Ter	P	nonsense
	c.11646_11659del	Ex42	p.Ser3883CysfsTer72	P	frameshift
	c.11705_11708del	Ex42	p.Thr3902ArgfsTer41	LP	frameshift
	c.11766G>A	Ex43	p.Trp3922Ter	P	missense
	c.11881C>T	Ex43	p.Gln3961Ter	P	nonsense
	c.11967_11974dup	Ex43	p.Ser3992TrpfsTer49	P	frameshift
	c.1198C>T	Ex5	p.Arg400Ter	P	nonsense
	c.12008dupA	Ex 44	p.Gln4005AlafsTer152	P	frameshift
	c.12010C>T	Ex44	p.Gln4004Ter	P	nonsense
	c.12058C>T	Ex44	p.Arg4021Ter	P	nonsense
	c.1273_1275del	Ex6	p.Glu425del	LP	in frame
	c.1987C>T	Ex10	p.Gln663Ter	P	nonsense
	c.2028dupC	Ex10	p.Gly677ArgfsTer37	LP	frameshift
	c.2085delC	Ex10	p.Ala696ArgfsTer89	P	frameshift
	c.2180T>C	Ex11	p.Leu727Pro	LP	missense
	c.2215dupC	Ex11	p.Gln739ProfsTer59	LP	frameshift
	c.2534T>C	Ex11	p.Leu845Ser	P	missense
	c.271_272delTC	Ex2	p.Ser91GlyfsTer22	P	frameshift
	c.2711_2712delAG	Ex11	p.Glu904GlyfsTer196	LP	frameshift
	c.3067C>T	Ex13	p.Gln1023Ter	P	nonsense
	c.3202C>T	Ex14	p.Gln1068Ter	P	nonsense
	c.3295+2T>C	Int14	-	LP/P	splicing
	c.3349C>T	Ex15	p.Gln1117Ter	P	nonsense
	c.3398_3399del	Ex15	p.Val1133GlufsTer2	P	frameshift
	c.3514C>T	Ex15	p.Gln1172Ter	P	nonsense
	c.3520_3527del	Ex15	p.Gln1174Cysfs34Ter	LP	frameshift
	c.359+2T>G	Int3	-	P	Splicing
	c.3706C>T	Ex15	p.Gln1236Ter	P	nonsense
	c.3745del	Ex15	p.Asp1249ThrfsTer24	P	frameshift
	c.3802C>T	Ex15	p.Gln1268Ter	LP	nonsense
	c.3955G>A	Ex15	p.Gly1319Arg	P	missense
	c.427C>T	Ex4	p.Gln143Ter	LP	nonsense
	c.4888C>T	Ex15	p.Gln1630Ter	P	nonsense
	c.4951C>T	Ex15	p.Gln1651Ter	P	nonsense
	c.5014_5015del	Ex15	p.Arg1672GlyfsTer98	P	frameshift
	c.5154_5163dup	Ex15	p.Met1722GlyfsTer52	P	frameshift

	c.5607dup	Ex15	p.Asn1870GlnfsTer120	P	frameshift
	c.5609A>G	Ex15	p.Asn1870Ser	LP	missense
	c.5869_5870dup	Ex15	p.Ser1957ArgfsTer16	LP	frameshift
	c.5884C>T	Ex15	p.Gln1962Ter	P	nonsense
	c.5905G>T	Ex15	p.Glu1969Ter	P	nonsense
	c.6050C>T	Ex15	p.Ser2017Leu	P	missense
	c.6199C>T	Ex15	p.Gln2067Ter	P	nonsense
	c.6504C>G	Ex15	p.Tyr2168Ter	P	nonsense
	c.6560G>A	Ex15	p.Trp2187*	P	nonsense
	c.680_681ins	Ex5	p.Gln227HisfsTer34	LP	frameshift
	c.6916-9G>A	Int15	-	LP	splicing
	c.7416_7417ins	Ex18	p.Gly2473ArgfsTer28	LP	frameshift
	c.7597_7598del	Ex19	p.Ser2533GlnfsTer61	P	missense
	c.7864_7899del	Ex21	p.Tyr2622_Lys2633del	p	in frame
	c.7984C>T	Ex21	p.Gln2662Ter	LP	nonsense
	c.8162-1G>A	Int22	-	P	splicing
	c.8238delG	Ex23	p.Met2747TrpfsTer9	LP	frameshift
	c.8279T>G	Ex23	p.Met2760Arg	LP	missense
	c.8284_8295del	Ex23	p.Ile2762_Arg2765del	LP	in frame
	c.8311G>A	Ex23	p.GlU2777 Lys	P	missense
	c.8371_8372dup	Ex23	p.Ser2792GlyfsTer84	P	frameshift
	c.8698C>T	Ex23	p.Gln2900Ter	P	nonsense
	c.8935_8937del	Ex24	p.Phe2979del	P	in frame
	c.9425_9426ins	Ex27	p.Tyr3143ValfsTer36	P	frameshift
	c.6504C>G	Ex15	p.Tyr2168Ter	P	nonsense
	c.9562A>G	Ex27	p.Asn3188Asp	LP	missense
	c.9564_9566del	Ex27	p.Asn3188del	LP	in frame
	c.9568G>C	Ex27	p.Gly3190Arg	P	missense
	c.9771_9774del	Ex29	p.Phe3257LeufsTer58	P	frameshift
**PKD2**	c.1094+3_1094+6del	Int4	-	P	splicing
	c.1142G>T	Ex5	p.Gly381Val	LP	missense
	c.1244T>G	Ex5	p.Leu415Arg	P	missense
	c.1249C>T	Ex5	p.Arg417Ter	P	nonsense
	c.1319+1G>A	Int5	-	P	splicing
	c.1395T>A	Ex6	p.Tyr465Ter	LP	nonsense
	c.1837C>T	Ex8	p.Gln613Ter	P	nonsense
	c.2117del	Ex10	p.Lys706ArgfsTer10	LP	frameshift
	c.2358del	Int 11	p.Glu787ArgfsTer14	P	splicing
	c.2419C>T	Ex13	p.Arg807Ter	P	nonsense
	c.2614 C>T	Ex14	p.Arg872Ter	LP	nonsense
	c.261G>A	Ex1	p.Trp87Ter	P	nonsense
	c.595+3A>T	Int1	-	P	Splicing
	c.637C>T	Ex2	p.Arg213Ter	P	nonsense
	c.709+1G>A	Int2	-	P	splicing
	c.916C>T	Ex4	p.Arg306Ter	P	nonsense
	c.958C>T	Ex14	p.Arg872Ter	P	nonsense
	c.964C>T	Ex4	p.Arg322Trp	P	missense
	c.974G>A	Ex4	p.Arg325Gln	LP	missense
VHL	c.217C>T	Ex1	p.Gln73Ter	P	nonsense
	c.598C>T	Ex3	p.Arg200Trp	P	missense
GANAB	c.2087G>A	Ex18	p.Arg696Gln	LP	missense
HNF1B	c.1462C>T	Ex7	p.Gln488Ter	LP	nonsense
UMOD	c.767G>A	Ex3	p.Cys256Tyr	LP	Mssense
TSC2	c.5176C>T	Ex40	p.Arg1726Trp	LP	missense
LRP5	c.1481G>A	Ex7	p.Arg494Gln	LP	missense
	c.3688G>T	Ex17	p.Ala649Ser	LP	missense

Supplementary Table S3: Pathogenic and likely pathogenic mutations in AR genes

	cDNA change	Region	Protein variant	ACMG Classification	
PKHD1	c.1690C>T	Ex18	p.Arg564Ter p	Pathogenic	nonsense
	c.9107T>G	Ex58	p.Val3036Gly	Likely Pathogenic	missense
	c.431C>T	Ex6	p.Pro144Leu	Likely Pathogenic	missense
PMM2	c.713G>A	Ex8	p.Arg238His	Pathogenic	missense
	c.422G>A	Ex5	p.Arg141His	Pathogenic	missense
CEP164	c.3055C>T	Ex24	p.Gln1019Ter	Pathogenic	nonsense
MAPKBP1	c.2271del	Ex19	p.Gly758AspfsTer74	Likely Pathogenic	Frameshift

Supplementary Table S4: VUS variants in AD genes

	cDNA change	Region	Protein variant	ACMG Classification	Coding impact
PKD1	c.11639_11683del	Ex42	p.Ala3880_Ala3894del	VUS	in frame
	c.12908A>T	Ex46	p.4303LeuextTer35	VUS	stopLoss
	c.9404C>T	ex27	p.Thr3135Met	VUS	missense
	c.2985+3A>C	Int12	-	VUS	splicing
	c.2189C>T	Ex11	p.Ser730Leu	VUS	missense
	c.12446G>A	Ex46	p.Arg4150His	VUS	missense
	c.9625C>T	Ex28	p.Arg3209Cys	VUS	missense
	c.5911G>A	Ex15	p.Val1971Met	VUS	missense
	c.11870G>A	Ex43	p.Gly3957Asp	VUS	missense
	c.11585T>G	Ex42	p.Leu3862Arg	VUS	missense
	c.4835C>T	Ex15	p.Thr1612Met	VUS	missense
	c.1832G>C	Ex9	p.Arg611Pro	VUS	missense
	c.10914G>T	Ex37	p.Val3638=; p.Thr1667Ala;	VUS	Synonimus;
	c.4999A>G	Ex15	p.Ser246Thr; p.Ala222Thr	VUS	missense;
	c.736T>A	Ex5		VUS	missense;
	c.664G>A	Ex5		VUS	missense
	c.3605C>T	Ex15	p.Ala1202Val	VUS	missense
	c.6484C>T	Ex15	p.Arg2162Trp	VUS	missense
	c.9676A>G	Ex28	p.Asn3226Asp	VUS	missense
PKD2	c.608C>T	Ex2	p.Thr203Ile	VUS	missense
	c.2670+5G>A	Int 14	-	VUS	Splicing
HNF1B	c.226G>T	Ex1	p.Gly76Cys	VUS	missense
ACTN4	c.59A>G	Ex1	p.Asn20Ser	VUS	missense
LRP5	c.226G>A	Ex2	p.Ala76Thr	VUS	missense
	c.713C>A	Ex43	p.Thr238Lys	VUS	missense

Supplementary Table S5: VUS alterations in AR genes

	cDNA change	Region	Protein variant	ACMG Classification	Coding impact
PKHD1	c.10036T> C	Exon 60	p.Cys3346Arg	VUS	missense
	c.5585C>T	Exon 34	p.Ser1862Leu	VUS	missense
	c.9402G>A	Exon 58	p.Lys3134=	VUS	synonymous
	c.5665G>A	Ex35	p.Glu1889Lys	VUS	missense
	c.11338C>T	Ex63	p.Pro3780Ser	VUS	missense
	c.10538G>A	Ex61	p.Ser3513Asn	VUS	missense
	c.129T>C	Ex3	p.Asp43=	VUS	synonymous
NPHS1	c.2746G>T	Ex20	p.Ala916Ser	VUS	missense
CEP290	c.4555A>T	Ex35	p.Ile1519Leu	VUS	missense
ZNF423	c.2657G>A	Ex43	p.Arg886Gln	VUS	missense
MAPKBP1	c.3776C>G	Ex30	p.Ser1259Cys	VUS	missense
MYO1E	c.1135C>G	Ex11	p.His379Asp	VUS	missense
CC2D2A	c.4409T>G	Ex35	p.Leu1470Arg	VUS	missense
NPHS2	c.686G>A	Ex5	p.Arg229Gln	VUS	missense
NEK8	c.1837A>T	Ex13	p.Ile613Phe	VUS	missense
NEK8	c.1736C>T	Ex13	p.Ser579Leu	VUS	missense
NPHP3	c.1930G>A	Ex13	p.Val644Met	VUS	missense
NPHS1	c.3418C>T	Ex27	p.Arg1140Cys	VUS	missense
NPHS1	c.2819G>T	Ex21	p.Arg940Leu	VUS	missense
BBS4	c.1027C>A	Ex12	p.Leu343Ile	VUS	missense
FOXI1	c.908G>A	Ex2	p.Gly303Glu	VUS	missense
ANKS6	c.1400G>A	Ex7	p.Arg467Gln	VUS	missense
COL4A4	c.1460G>A	Ex22	p.Gly487Glu	VUS	missense
INVS	c.2296C>A	Ex15	p.Leu766Met	VUS	missense
TTC21B	c.667C>G	Ex6	p.Leu223Val	VUS	missense
CD2AP	c.491A>T	Ex5	p.Glu164Val	VUS	missense
